# Geographical Provenance-Associated Differences in Lipophilic Phytochemical Fingerprints and Biological Activities of *Peltophorum africanum* from Two South African Collection Sites

**DOI:** 10.3390/life16101580

**Published:** 2026-09-22

**Authors:** Mahlogonolo Phasha, Vusumuzi Mabasa, Mmamosheledi E. Mothibe, David N. Nogbou, Andrew M. Musyoki, Sechene S. Gololo, Hlayiseka Shikwambana, Ntwanano S. Mapfumari

**Affiliations:** 1 Department of Biochemistry and Biotechnology, School of Science and Technology, Sefako Makgatho Health Sciences University, Pretoria 0182, South Africa; mahlogonolophasha24@gmail.com (M.P.); stanley.gololo@smu.ac.za (S.S.G.); hlayiseka.shikwambana@smu.ac.za (H.S.); 2 Division of Epidemiology & Biostatistics, School of Public Health, Faculty of Health Sciences, University of the Witwatersrand (Wits), Johannesburg 2001, South Africa; mabasavusumuzi@gmail.com; 3 Division of Pharmacology, Faculty of Pharmacy, Rhodes University, Makhanda 6139, South Africa; mamza.mothibe@ru.ac.za; 4 Department of Medical Microbiology, School of Medicine, Sefako Makgatho Health Sciences University, Pretoria 0182, South Africa; davnogbou2@gmail.com (D.N.N.); andrew.musyoki@smu.ac.za (A.M.M.); 5 Department of Physiology, School of Medicine, Sefako Makgatho Health Sciences University, Pretoria 0182, South Africa

**Keywords:** *Peltophorum africanum*, geographical provenance, lipophilic extracts, phytochemical fingerprint, antioxidant activity, antibacterial activity, thin-layer chromatography

## Abstract

Medicinal plants collected from different environments may yield chemically and biologically distinct preparations even when they share the same botanical identity. This study compared the lipophilic phytochemical profiles and in vitro antioxidant and antibacterial activities of *Peltophorum africanum* leaf material collected at one purposively selected site in Bushbuckridge, Mpumalanga (MP), and one in Mafarana Village, Limpopo (LP), South Africa. Hexane and dichloromethane (DCM) extracts were examined using qualitative and quantitative phytochemical assays, thin-layer chromatography (TLC), DPPH-TLC bioautography, three antioxidant assays and antibacterial screening. The four site-derived extracts showed different phytochemical and chromatographic patterns. The MP hexane extract had the lowest DPPH IC50 (0.19 mg/mL), whereas the LP DCM extract had the lowest hydrogen peroxide-scavenging IC50 (0.48 mg/mL). Ferric reducing power IC50 values were similar across the extracts (0.48–0.55 mg/mL). In broth microdilution, only the DCM extracts inhibited *Enterococcus faecalis* at the tested range (MIC = 0.50 mg/mL for both sites); all other extract-organism combinations had MIC values > 1.00 mg/mL. The findings demonstrate geographical provenance-associated variation between the two sampled localities and identify assay-specific candidates for further study. They do not, however, establish that either single site represents its entire province. Broader multi-site and multi-season sampling with compound-level characterisation would strengthen geographical generalisation and mechanistic interpretation.

## 1. Introduction

Medicinal plants remain indispensable sources of therapeutic agents and continue to contribute to primary healthcare and natural-product drug discovery, particularly in communities where traditional medicine is culturally embedded and readily accessible [1,2,3,4,5,6,7,8]. Their therapeutic properties arise from structurally diverse specialised metabolites, including alkaloids, flavonoids, terpenoids, cardiac glycosides, phenolic compounds, tannins and steroids [9,10,11,12,13]. These constituents may act individually or in combination and have been associated with antioxidant, antimicrobial, anti-inflammatory and anticancer activities [14,15,16,17,18]. Chemical consistency is therefore central to the reproducibility, quality assurance and pharmacological development of medicinal-plant preparations.

Botanical identity alone does not ensure phytochemical uniformity. Secondary-metabolite biosynthesis and accumulation may respond to temperature, rainfall, altitude, soil characteristics, solar exposure and water availability [19,20,21]. Plants of the same species collected from different environments can consequently show different chemical profiles and biological responses [22,23]. These observations support geographically informed comparison, while also underscoring the need to distinguish observed site-level differences from province-wide generalisation.

Among specialised metabolites, lipophilic constituents are of interest because terpenoids, steroids, lipophilic flavonoids and cardiac glycosides can contribute to antioxidant, antibacterial, anti-inflammatory and other pharmacological effects [13,24,25]. Hexane and dichloromethane preferentially recover relatively non-polar compounds that may be underrepresented in aqueous or strongly polar preparations and can provide useful leads during bioactivity-guided investigation [26,27,28]. Polar extracts may yield additional or stronger activities, but activity maximisation across every solvent class was not the objective of this work; the study deliberately addressed the less frequently characterised lipophilic chemical space.

*Peltophorum africanum* Sond. (Fabaceae) is an indigenous southern African medicinal tree used traditionally for bacterial infections, inflammatory disorders, pain and conditions associated with oxidative stress [29,30,31]. Previous investigations have reported antioxidant, antibacterial, antifungal, anti-inflammatory and antiproliferative activities in extracts of the species [32,33,34]. Although *P. africanum* has been studied in other South African settings, direct comparison of its lipophilic leaf extracts from Limpopo and Mpumalanga remained limited. This gap is relevant because communities may use the same species differently and because collection provenance can influence the reproducibility of plant-derived preparations [35,36,37,38,39].

The Limpopo and Mpumalanga sites were selected purposively because they represented two distinct community-use contexts known to the research team and the participating Indigenous Knowledge holders. The objective was to compare the phytochemical composition, TLC fingerprints, antioxidant activity and antibacterial activity of hexane and dichloromethane leaf extracts prepared from the two provenances. We hypothesised that the contrasting collection localities would yield distinguishable lipophilic profiles and bioactivities. The inference is intentionally restricted to the sampled provenances; the study does not claim that one locality represents all *P. africanum* populations within its province.

## 2. Materials and Methods

### 2.1. Study Design

This comparative experimental study examined *P. africanum* leaf material from two purposively selected geographical provenances: Bushbuckridge in Mpumalanga and Mafarana Village in Limpopo. The selection was ethnobotanically motivated by differing community-use contexts associated with the two localities. Hexane and dichloromethane extracts from each pooled site sample were compared by phytochemical assays, TLC fingerprinting, antioxidant assays and antibacterial assays. The design permits comparison of the sampled provenances, but not statistical representation of all populations within either province. Assay triplicates were technical measurements of each site-derived extract.

### 2.2. Plant Collection, Authentication and Preparation

Fresh leaves of *P. africanum* were collected from naturally growing trees at one locality in Bushbuckridge, Mpumalanga Province (24.8398° S, 31.0464° E), and one locality in Mafarana Village, Limpopo Province (23.9649° S, 30.3803° E), South Africa, during September 2020 with the assistance of an Indigenous Knowledge practitioner. The number of individual trees contributing at each locality was not recorded. Leaves collected within each site were pooled before processing to form one composite sample per provenance. The plant material was authenticated at the South African National Biodiversity Institute (SANBI), and a voucher specimen (MC001) was deposited in the Herbarium of the Pharmaceutical Sciences Unit, School of Pharmacy, Sefako Makgatho Health Sciences University. The two pooled samples were processed separately under identical laboratory conditions. Leaves were air-dried at room temperature in a well-ventilated room for two weeks, ground using a Polymix PX-MFC 90 D grinder (Kinematic AG, Luzern, Switzerland) and stored in airtight glass containers at room temperature until extraction.

### 2.3. Preparation of Non-Polar Extracts

Sequential solvent extraction was performed according to Gololo et al. [39] and Mapfumari et al. [9], with minor modifications. Approximately 250 g of powdered leaf material from each site was extracted successively with n-hexane and then dichloromethane to target lipophilic constituents. Each solvent extraction was conducted for 24 h and repeated for three consecutive extraction cycles with intermittent agitation to maximise recovery. The filtrates were obtained using qualitative filter paper (Boeco Grade 3 HW, 125 mm; BOECO Germany GmbH, Hamburg, Germany), concentrated under reduced pressure at 37 °C and 50 rpm, air-dried to remove residual solvent, transferred into labelled airtight containers and stored at 4 °C until analysis.

### 2.4. Qualitative Phytochemical Screening

Qualitative phytochemical screening was performed according to previously described procedures [39,40], with minor modifications, to compare the two site-derived samples.

#### 2.4.1. Alkaloids

For alkaloids, 5 mL of crude extract dissolved in the corresponding extraction solvent was treated with 10 drops of Dragendorff’s reagent. Formation of an orange-red precipitate was interpreted as a positive reaction [39,40].

#### 2.4.2. Anthraquinones

For anthraquinones, 1 mL of crude extract was treated with five drops of 10% ammonia. Development of a pink or reddish colour was interpreted as a positive reaction [39,40].

#### 2.4.3. Cardiac Glycosides

For cardiac glycosides, 2 mL of crude extract was mixed with 1 mL of glacial acetic acid and 1 mL of ferric chloride, followed by the careful addition of 1 mL of concentrated sulphuric acid. Development of a green or blue colour was interpreted as a positive reaction [39,40].

#### 2.4.4. Saponins

For saponins, 0.5 mL of crude extract was mixed with 5 mL of water and vortexed. Persistent foam formation was interpreted as a positive reaction [39,40].

#### 2.4.5. Phenolic Compounds

For phenolic compounds, 2 mL of crude extract was treated with 0.5 mL of ferric chloride. Development of an intense colour was interpreted as a positive reaction [39,40].

#### 2.4.6. Tannins

For tannins, 2 mL of crude extract was mixed with 1 mL of ferric chloride. Development of a green, blue or brown colour was interpreted as a positive reaction [39,40].

#### 2.4.7. Terpenoids

For terpenoids, the Salkowski test was used: 5 mL of crude extract was mixed with 2 mL of chloroform and 3 mL of concentrated sulphuric acid. Development of a reddish-brown colour was interpreted as a positive reaction [39,40].

#### 2.4.8. Flavonoids

For flavonoids, 1 mL of diluted crude extract was mixed with 1 mL of dilute sodium hydroxide, followed by 1 mL of dilute hydrochloric acid. A yellow colour after sodium hydroxide addition that became colourless after hydrochloric acid addition was interpreted as a positive reaction [39,40].

#### 2.4.9. Steroids

For steroids, 2 mL of crude extract was mixed with 2 mL of chloroform, after which 1 mL of sulphuric acid was added slowly. Formation of a red lower layer was interpreted as a positive reaction [39,40].

### 2.5. Quantitative Determination of Lipophilic Phytochemicals

Flavonoid, phenolic, tannin and saponin contents were determined using previously described spectrophotometric methods [9,39]. Total flavonoid content was measured using the aluminium chloride colourimetric assay and expressed as milligrams of quercetin equivalents per gram of extract (mg QE/g extract). Total phenolic and tannin contents were measured using Folin–Ciocalteu chemistry and expressed as milligrams of gallic acid equivalents per gram of extract (mg GAE/g extract). Total saponin content was measured using the vanillin-sulphuric acid assay and expressed as milligrams of diosgenin equivalents per gram of extract (mg DE/g extract). Each assay was performed in technical triplicate on the corresponding site-derived extract.

#### 2.5.1. Determination of Total Tannin Content

Total tannin content was determined using the Folin–Ciocalteu method [9,39]. Gallic acid standards (20, 40, 60, 80 and 100 mg/mL) were prepared for the standard curve. A 0.5 mL volume of each standard or extract sample was combined with 2.5 mL of Folin–Ciocalteu reagent and 1 mL of 35% Na_2_CO_3_ in a 50 mL volumetric flask, made to volume with distilled water, mixed and incubated at room temperature for 30 min. Absorbance was measured at 725 nm using a UV-visible spectrophotometer (Spectroline CM-10A, Westbury, NY, USA). Extracts were measured in technical triplicate, and results were expressed as mg GAE/g extract.

#### 2.5.2. Determination of Total Phenolic Content

The total phenolic content was determined using the Folin–Ciocalteu assay, as described by [9,39]. Standard solutions of gallic acid (20, 40, 60, 80, 100 mg/mL) were prepared in triplicate, from which 1 mL of each of the gallic acid solutions was added to a 25 mL volumetric flask to create a reaction mixture. Then, 1 mL of Folin–Ciocalteu phenol reagent was added. After 5 min, 10 mL of 7% Sodium carbonate solution was added to the mixture, and the volume was made up to 25 mL with distilled water. The solution was then incubated for 90 min at room temperature, and the absorbance was read at 550 nm with an ultraviolet/visible (UV/vis) spectrophotometer (Spectroline, model CM–10A, Fluorescence analysis cabinet, Westbury, New York, NY, USA). Samples (1 mL) of the crude extracts of thetwo site-derived samples were prepared in triplicate and taken through the procedure described above, and their absorbance was read following the same process. The total phenol content of the extracts was expressed as mg gallic acid equivalence/g of extract (mg GAE/g extract).

#### 2.5.3. Determination of Total Flavonoid Content

The total flavonoid content was determined using the aluminium chloride colourimetric assay, as described by [9,39]. Standard solutions of quercetin (20, 40, 60, 80, and 100 mg/mL) were prepared in triplicate, from which 1 mL of each concentration was taken and used to create a reaction mixture. The reaction mixture consisted of 1 mL of sample, 3 mL of methanol, 0.2 mL of 10% aluminium chloride, 0.2 mL of 1M potassium acetate, and 5.6 mL of distilled water. The reaction mixture was then left at room temperature for 30 min, and then the absorbance was read at 420 nm using a UV/vis spectrophotometer (Spectroline, model CM–10A, Fluorescence analysis cabinet, Westbury, New York, NY, USA). Samples (1 mL) of the crude extracts of thetwo site-derived samples were prepared in triplicate and taken through the procedure described above, and their absorbance was read following the same process. The total flavonoid content was expressed as mg quercetin equivalence/g of extract (mg QE/g extract).

#### 2.5.4. Determination of Total Saponin Content

The total saponin content was determined using the vanillin-sulphuric acid colourimetric reaction, as described by [9,39]. Standard solutions of diosgenin (20, 40, 60, 80, and 100 mg/mL) were prepared in triplicate, from which 50 µL of each concentration was diluted with 250 µL of distilled water. To this, about 250 µL of vanillin reagent (800 mg of vanillin in 10 mL of 99.5% ethanol) was added. Then 2.5 mL of 72% sulphuric acid was added, and the mixture was mixed well. This solution was kept in a water bath at 60 °C for 10 min. After 10 min, it was cooled in ice-cold water, and the absorbance was read at 544 nm with a UV/vis spectrophotometer (Spectroline, model CM–10A, Fluorescence analysis cabinet, Westbury, New York, NY, USA). Samples (50 µL) of the crude extracts of the two site-derived samples were prepared in triplicate and taken through the procedure described above, and their absorbance was read following the same process. The values were expressed as mg diosgenin equivalents/g extract (mg DE/gextract) derived from a standard curve.

### 2.6. Thin-Layer Chromatographic Fingerprinting

Thin-layer chromatography (TLC) was performed to compare the chemical fingerprints of the non-polar extracts as previously described by Mapfumari et al. [9], with minor modifications. The hexane and dichloromethane extracts obtained from the Mpumalanga and Limpopo samples were applied separately near the base of silica gel 60 F254 TLC plates (Merck, Darmstadt, Germany). The plates were developed in a chromatographic chamber containing hexane:ethyl acetate (8.5:2.5, *v*/*v*) as the mobile phase. Development was allowed to proceed until the solvent front had migrated approximately 8 cm from the point of application.

Following development, the TLC plates were removed from the chamber and air-dried to allow complete evaporation of the mobile phase. The chromatograms were initially examined under visible light and subsequently under ultraviolet illumination at 254 and 365 nm. The plates were then derivatised by spraying with freshly prepared vanillin-sulphuric acid reagent and heated at 105 °C until coloured bands became visible. The chromatographic profiles of the Mpumalanga and Limpopo extracts were compared according to the number, colour, intensity and migration patterns of the resolved bands. Retention factor (Rf) values were calculated as the distance travelled by an individual compound band divided by the distance travelled by the solvent front.

### 2.7. Qualitative Antioxidant Screening

#### 2.7.1. Dot-Plot Assay

The presence of antioxidant constituents in the non-polar extracts was initially evaluated using the DPPH dot-plot assay as previously described [9,10], with minor modifications. Small aliquots of each hexane and dichloromethane extract were applied directly onto silica gel TLC plates and allowed to air-dry completely. The plates were uniformly sprayed with freshly prepared 0.2% (*w*/*v*) 2,2-diphenyl-1-picrylhydrazyl (DPPH) solution in methanol.

Following application of the DPPH reagent, the plates were maintained in darkness to minimise light-induced degradation of the radical. Antioxidant activity was identified by the appearance of yellow or pale areas against the purple DPPH background. The intensity of the discolouration was used as a qualitative indication of the relative radical-scavenging activity of the extracts.

#### 2.7.2. TLC-DPPH Bioautography

Thin-layer chromatography coupled with DPPH bioautography was used to separate and localise antioxidant-active constituents within the non-polar extracts [10,41]. The hexane and dichloromethane extracts from Mpumalanga and Limpopo were applied separately onto silica gel 60 F254 TLC plates and developed using hexane:ethyl acetate (8.5:2.5, *v*/*v*) as the mobile phase. Development was continued until the solvent front had migrated approximately 8 cm from the point of application.

The developed plates were removed from the chamber, air-dried and examined under visible and ultraviolet light before being sprayed uniformly with freshly prepared 0.2% DPPH solution in methanol. The plates were subsequently incubated in darkness until colour development was complete. Antioxidant-active constituents appeared as yellow or pale bands against the purple chromatographic background. The number, relative intensity, distribution and Rf values of the antioxidant-active bands were recorded and compared between the Mpumalanga and Limpopo extracts.

### 2.8. Quantitative Antioxidant Activity

The antioxidant activity of the non-polar extracts was evaluated using three complementary in vitro assays: DPPH radical-scavenging, hydrogen peroxide scavenging, and ferric reducing power assays. Extracts were evaluated at concentrations ranging from 0.2 to 1.0 mg/mL. Gallic acid and butylated hydroxytoluene (BHT) were included as reference antioxidants. All assays were performed in triplicate.

#### 2.8.1. DPPH Radical-Scavenging Assay

The DPPH radical-scavenging activity of the extracts was evaluated according to previously described methods [9,10], with minor modifications. Hexane and dichloromethane extract solutions were prepared at concentrations of 0.2, 0.4, 0.6, 0.8 and 1.0 mg/mL. A volume of 1 mL of each extract concentration was mixed with 1 mL of freshly prepared 0.2 mM DPPH solution in methanol. The mixtures were vortexed briefly and incubated in darkness at room temperature for 30 min to allow completion of the radical-scavenging reaction. Absorbance was measured at 517 nm using a UV/visible spectrophotometer. A control containing DPPH solution without plant extract was prepared under identical conditions. Gallic acid and BHT were evaluated using the same procedure. The percentage DPPH radical-scavenging activity was calculated as follows:

$$\mathrm{DPPH}\;\mathrm{inhibition}\left(\%\right)=\left(\frac{A_{control}-A_{sample}}{A_{control}}\right)\times 100$$

where *A*_control_ represents the absorbance of the DPPH solution without extract, and *A*_sample_ represents the absorbance of the reaction mixture containing the extract or reference antioxidant. The concentration required to inhibit 50% of the DPPH radicals (IC_50_) was determined from the concentration–response curves.

#### 2.8.2. Hydrogen Peroxide Scavenging Assay

Hydrogen peroxide scavenging activity was determined using a modified method described previously [9,10]. Extract solutions were prepared at concentrations of 0.2, 0.4, 0.6, 0.8 and 1.0 mg/mL. A volume of 1 mL of each extract concentration was mixed with 2 mL of 20 mM hydrogen peroxide solution prepared in phosphate-buffered saline at pH 7.4. The reaction mixtures were incubated at room temperature for 10 min. Absorbance was subsequently measured at 230 nm against a corresponding blank containing phosphate-buffered saline without hydrogen peroxide. Gallic acid and BHT were evaluated under the same experimental conditions. Hydrogen peroxide scavenging activity was calculated relative to the hydrogen peroxide control, and IC_50_ values were determined from the concentration–response curves. The percentage hydrogen peroxide scavenging activity was calculated using the following equation:

$$\mathrm{Hydrogen}\;\mathrm{peroxide}\;\mathrm{scavenging}\;\mathrm{activity}\left(\%\right)\left(\frac{A_{control}-A_{sample}}{A_{control}}\right)\times \;100$$

where *A*_control_ represents the absorbance of the hydrogen peroxide solution without extract and *A*_sample_ represents the absorbance in the presence of the extract or reference antioxidant.

#### 2.8.3. Ferric Reducing Power Assay

The reducing power of the extracts was determined according to the method described previously [9,39], with minor modifications. Extract solutions were prepared at concentrations ranging from 0.2 to 1.0 mg/mL. A volume of 2 mL of each extract concentration was mixed with 2.5 mL of 0.2 M phosphate buffer at pH 6.6 and 2.5 mL of 1% potassium ferricyanide solution. The mixtures were incubated at 50 °C for 20 min.

Following incubation, 2.5 mL of 10% trichloroacetic acid was added to each mixture to terminate the reaction. The mixtures were centrifuged at 3000 rpm for 10 min. Thereafter, 2.5 mL of the resulting supernatant was mixed with 2.5 mL of distilled water and 0.5 mL of 0.1% ferric chloride solution. Absorbance was measured at 700 nm using a UV/visible spectrophotometer. Increased absorbance was interpreted as increased electron-donating and ferric-reducing capacity. Gallic acid and BHT were evaluated under the same conditions as reference antioxidants.

### 2.9. Antibacterial Activity

#### 2.9.1. Test Microorganisms

The extracts were evaluated against two Gram-negative bacteria, *Escherichia coli* ATCC 35716 and *Acinetobacter baumannii* ATCC 19606, and two Gram-positive bacteria, *Staphylococcus aureus* ATCC 25923 and *Enterococcus faecalis* ATCC 51299.

The bacterial organisms were cultured in Mueller–Hinton broth and incubated overnight at 37 °C. Before use in the antibacterial assays, the turbidity of each bacterial suspension was adjusted to a 0.5 McFarland standard, corresponding to approximately 1.5 × 10^8^ colony-forming units per millilitre (CFU/mL). Standardised inocula were used for the qualitative dot plot, TLC bioautography and broth microdilution assays.

#### 2.9.2. Qualitative Antibacterial Dot-Plot Assay

Preliminary antibacterial activity of the non-polar extracts was evaluated using a qualitative dot-plot assay, adapted from previously described antibacterial screening procedures [9,42,43]. The hexane and dichloromethane extracts obtained from *Peltophorum africanum* collected in Mpumalanga and Limpopo Provinces were prepared at a concentration of 1 mg/mL in their respective extraction solvents. Aliquots of the extract solutions were applied as discrete spots onto Mueller–Hinton agar plates previously inoculated with standardised suspensions of *Escherichia coli* ATCC 35716, *Acinetobacter baumannii* ATCC 19606, *Staphylococcus aureus* ATCC 25923 and *Enterococcus faecalis* ATCC 51299.

The bacterial suspensions were prepared from overnight cultures and adjusted to a 0.5 McFarland turbidity standard, corresponding to approximately 1.5 × 10^8^ CFU/mL. The inoculated plates were incubated at 37 °C for 24 h. Following incubation, antibacterial activity was evaluated qualitatively by observing clear areas of growth inhibition surrounding the extract application sites. The presence of a clear inhibition area was interpreted as evidence of antibacterial activity, while uninterrupted bacterial growth surrounding an extract spot was interpreted as absence of detectable activity under the assay conditions. The qualitative dot-plot assay was used as an initial antibacterial screen and was complemented by TLC bioautography and broth microdilution MIC determination.

#### 2.9.3. TLC Bioautography

TLC bioautography was performed to localise antibacterial-active constituents within the hexane and dichloromethane extracts according to previously described methods [9,43], with minor modifications. The extracts were applied approximately 1.5 cm from the base of silica gel 60 F254 TLC plates. The plates were developed using hexane:ethyl acetate (8.5:2.5, *v*/*v*) until the solvent front had migrated at least 8 cm from the base.

Following chromatographic development, the plates were removed from the chamber and thoroughly air-dried to eliminate residual organic solvents. Each bacterial organism was cultured overnight in Mueller–Hinton broth, and the bacterial suspension was adjusted to a 0.5 McFarland standard, corresponding to approximately 1.5 × 10^8^ CFU/mL. The developed TLC plates were sprayed with the standardised bacterial suspension until uniformly wet. All inoculation procedures were conducted aseptically in a laminar-flow cabinet.

The inoculated plates were incubated overnight at 37 °C in darkness under conditions of approximately 100% relative humidity. Following incubation, the plates were sprayed with 2.0 mg/mL *p*-iodonitrotetrazolium violet (INT) solution and incubated further to allow visualisation of bacterial growth. Areas of bacterial growth developed a purple colour owing to the reduction in INT, whereas white to yellowish zones against the purple background indicated inhibition of bacterial growth. The positions and Rf values of all antibacterial-active bands were recorded and compared between extracts from Mpumalanga and Limpopo.

#### 2.9.4. Minimum Inhibitory Concentration (MIC)

Minimum inhibitory concentrations were determined using the broth microdilution method described previously [9,43]. The hexane and dichloromethane extracts were prepared at an initial concentration of 1.0 mg/mL. A volume of 150 µL of Mueller–Hinton broth was added to each well of sterile 96-well microtitre plates. Thereafter, 150 µL of each plant extract was added to the first well of the corresponding row and serially diluted two-fold across the plate to produce decreasing extract concentrations.

A volume of 50 µL of the standardised bacterial suspension, adjusted to approximately 1.5 × 10^8^ CFU/mL, was added to each test well. The plates were incubated overnight at 37 °C. Following incubation, two drops of 0.2 mg/mL INT solution were added to each well as a bacterial growth indicator.

Development of a pink or purple colour indicated bacterial metabolic activity, whereas the absence of colour development indicated inhibition of bacterial growth. The MIC was defined as the lowest concentration of extract at which no visible bacterial growth or INT colour development was observed. Where bacterial growth was still detected at the highest concentration tested, the MIC was reported as >1.00 mg/mL.

### 2.10. Data Analysis and Scope of Inference

Technical replicate measurements are summarised as mean ± standard deviation (SD) where replicate-level values were available. IC50 values were estimated from the concentration-response data and are reported as point estimates. The single collection site per province was the site-level experimental unit; therefore, technical replicates did not constitute independent biological replicates. Province-level hypothesis tests, mixed-effects models, correlation analysis and chemometric classification were not performed because the sampling design did not provide sufficient independent units. Comparisons between the two site-derived extracts are consequently descriptive and exploratory.

## 3. Results

### 3.1. Geographical Provenance-Associated Variation in Qualitative Phytochemical Profiles

Qualitative screening showed that the hexane and dichloromethane extracts prepared from the two geographical provenances shared several phytochemical classes, with site- and solvent-associated differences in selected reactions (Table 1).

Alkaloids were detected in both hexane extracts but not in either dichloromethane extract. Anthraquinones and steroids were not detected in any extract. Cardiac glycosides were detected in the MP hexane extract and in both dichloromethane extracts, with a stronger reaction in MP DCM than LP DCM. Flavonoids were detected in both hexane extracts and at trace levels in MP DCM, whereas phenolic compounds were detected only in the DCM extracts and tannins were detected at trace levels in both DCM extracts. Within the defined two-site comparison, these patterns demonstrate provenance-associated variation in the tested lipophilic extracts.

### 3.2. Geographical Provenance-Associated Variation in Quantitative Phytochemical Profiles

The technical replicate summaries showed numerical differences between the four extracts (Table 2). These values are presented descriptively because independent site-level biological replication was not available.

Flavonoid content was numerically higher in the MP than the LP extract for both solvents (hexane: 223.45 ± 16.74 vs. 136.00 ± 4.93 mg QE/g extract; DCM: 193.53 ± 88.72 vs. 155.12 ± 15.27 mg QE/g extract). The comparatively large SD for the MP DCM technical measurements indicates substantial assay variability and warrants cautious interpretation.

Saponin-equivalent measurements were much higher in the hexane than DCM extracts and were numerically higher for LP than MP within each solvent. This quantitative colour reaction should not be interpreted as confirmation of a purified saponin identity, particularly because the qualitative screening did not report saponins in Table 1.

Phenolic compounds were not detected quantitatively in any extract. Tannin-equivalent values ranged from 1.54 ± 0.33 to 3.74 ± 0.77 mg GAE/g extract. No inferential claim about differences between sites is made.

Overall, the measurements show location-associated numerical variation in the sampled material. This interpretation is consistent with earlier chromatographic and quantitative comparisons of medicinal plants collected from different South African localities [38,39], while remaining bounded to the two provenances studied here.

### 3.3. TLC Fingerprints Distinguish the Two Sampled Geographical Provenances

TLC showed differences in band number, intensity and Rf values among the four site-derived extracts (Figure 1 and Figure 2). These visual fingerprints distinguish the tested samples descriptively and support provenance-associated chemical variation within the scope of this comparison.

#### 3.3.1. Hexane Extracts

The hexane extracts produced well-resolved chromatographic profiles following development in the hexane:ethyl acetate (8.5:2.5, *v*/*v*) solvent system. Examination under visible light, ultraviolet illumination (254 and 365 nm) and after derivatisation with vanillin-sulphuric acid revealed multiple-coloured bands corresponding to chemically distinct lipophilic constituents (Figure 1).

Both site-derived hexane extracts shared prominent bands, while selected bands differed in presence or staining intensity. Vanillin-sulphuric acid colour and UV fluorescence provide only tentative class-level information; they do not identify individual compounds. LC-MS, GC-MS or NMR confirmation would be required to assign chemical identities.

#### 3.3.2. Dichloromethane Extracts

The DCM extracts also showed differences in band presence and intensity (Figure 2). Their broader band distribution relative to the hexane extracts is consistent with the intermediate polarity of DCM and its recovery of a different range of constituents.

Several bands were common to both DCM extracts, while others differed in presence or relative staining intensity. These differences distinguish the two tested extracts descriptively.

The TLC results therefore demonstrate distinguishable fingerprints for the two sampled provenances, but they are not presented as a validated classifier for unknown samples from the broader provinces.

Table 3 summarises selected bands that differed in presence, Rf or colour reaction between the tested site-derived extracts. These features are sufficient for a descriptive TLC comparison. PCA, PLS or another validated chemometric classifier would require a larger set of independent samples and was therefore neither appropriate nor necessary for the stated two-provenance objective.

### 3.4. Comparative Antioxidant Activity of the Extracts

#### 3.4.1. Qualitative Dot-Plot Screening

DPPH dot-plot screening indicated radical-scavenging constituents in the hexane and DCM extracts from both collection sites (Figure 3).

All extracts produced yellow discolouration against the purple DPPH background. Differences in colour intensity were treated as qualitative observations only.

The MP hexane spot showed the most intense discolouration. This observation was consistent with its lower DPPH IC50, but the qualitative assay does not quantify effect size or establish a geographical cause.

#### 3.4.2. TLC-DPPH Bioautography

TLC-DPPH bioautography localised radical-scavenging activity to several chromatographic bands in both solvent extracts (Figure 4). Band number, distribution and intensity differed between the two site-derived samples.

The MP hexane extract showed more prominent antioxidant-active bands than the LP hexane extract, while differences between the DCM extracts were less marked. These observations are qualitative.

Co-localisation of active zones with TLC bands identifies regions for future bioassay-guided isolation but does not establish individual compound identities.

#### 3.4.3. Quantitative Antioxidant Activity

The antioxidant assays produced an assay-dependent pattern (Table 4; Figure 5, Figure 6 and Figure 7). The MP hexane extract had the lowest DPPH IC50, whereas the LP DCM extract had the lowest hydrogen peroxide-scavenging IC50. Reducing power IC50 values were similar across the four extracts. Thus, no collection site was uniformly superior across antioxidant mechanisms.

##### DPPH Radical-Scavenging Activity

All extracts showed concentration-related DPPH scavenging over the tested range (Figure 5). The MP hexane extract had the lowest IC50 (0.19 mg/mL), followed by LP hexane (0.24 mg/mL), LP DCM (0.74 mg/mL) and MP DCM (0.81 mg/mL).

Within the DPPH assay, the hexane extracts were more active than the DCM extracts, and the MP hexane extract yielded the lowest IC50.

The numerical agreement between the MP hexane DPPH response, its flavonoid-equivalent measurement and its TLC-DPPH pattern is hypothesis-generating only; a correlation cannot be estimated validly from this design.

##### Hydrogen Peroxide Scavenging Activity

Hydrogen peroxide-scavenging responses differed from the DPPH ranking (Figure 6). The DCM extracts reached IC50 values within the tested range, with LP DCM (0.48 mg/mL) slightly lower than MP DCM (0.52 mg/mL). The hexane extracts did not reach 50% inhibition within 0.2–1.0 mg/mL; extrapolated values were 1.10 mg/mL for LP and 1.26 mg/mL for MP.

##### Ferric Reducing Power

Reducing power increased with extract concentration (Figure 7). The four IC50 estimates were tightly grouped (0.48–0.55 mg/mL): LP hexane, 0.48; MP hexane, 0.50; MP DCM, 0.54; and LP DCM, 0.55 mg/mL.

The differing rankings across DPPH, hydrogen peroxide and reducing power assays indicate assay- and solvent-dependent behaviour rather than a uniform site effect.

### 3.5. Comparative Antibacterial Activity of the Extracts

#### 3.5.1. Qualitative Antibacterial Dot-Plot Screening

Qualitative dot-plot screening was performed against *Staphylococcus aureus*, *Enterococcus faecalis*, *Escherichia coli* and *Acinetobacter baumannii* (Figure 8). Differences in appearance among extract-organism combinations were used only as a preliminary screen and were interpreted together with broth microdilution.

#### 3.5.2. TLC Bioautography Localises Antibacterial-Active Zones

Developed chromatograms were subjected to antibacterial bioautography using p-iodonitrotetrazolium violet (INT) as a growth indicator (Figure 9). Clear zones occurred at selected chromatographic positions in the hexane and DCM lanes. Their distribution varied among the tested extracts and organisms, indicating chromatographic regions for future isolation work.

Because no compounds were isolated or structurally characterised, the active zones cannot be assigned to individual phytochemicals. Differences in band appearance also cannot be used to infer a province-level effect.

These qualitative observations were therefore treated as screening evidence and evaluated against the MIC results.

#### 3.5.3. Minimum Inhibitory Concentrations

Broth microdilution showed measurable inhibition only for the DCM extracts against *E. faecalis*, with an MIC of 0.50 mg/mL for both collection sites (Table 5).

All hexane extract-organism combinations and all remaining DCM combinations had MIC values > 1.00 mg/mL. Accordingly, the MIC data do not support a difference in antibacterial potency between the two collection sites within the tested concentration range.

The apparent zones in the qualitative assays should therefore not be interpreted as quantitative evidence of site-specific antibacterial superiority.

Across the four extracts, the phytochemical assays, TLC and antioxidant assays demonstrated solvent- and provenance-associated patterns, whereas the MIC assay showed the same measurable result for both DCM extracts against *E. faecalis*. The combined evidence supports a geographically informed comparison of the sampled materials and defines specific hypotheses for broader follow-up.

## 4. Discussion

This study compared hexane and DCM leaf extracts prepared from pooled *P. africanum* material collected at Bushbuckridge and Mafarana Village. The extracts differed in qualitative phytochemical reactions, quantitative measurements, TLC bands and antioxidant responses. These differences constitute evidence of geographical provenance-associated variation between the two sampled localities. This interpretation is supported by previous work on *S. italica* from four Limpopo districts, in which TLC, UV-visible and quantitative phytochemical approaches identified collection-area differences [38,39], as well as by broader reports of geographical variation in medicinal plants [22,23,35,36,37,38,44]. The present claim is deliberately site-specific: it does not assert that either pooled sample represents every *P. africanum* population within Mpumalanga or Limpopo.

The TLC fingerprints showed shared and provenance-specific bands. TLC remains a recognised and accessible method for comparative fingerprinting and bioautography, particularly when the claim is limited to band patterns, Rf values and activity-localised regions. Vanillin-sulphuric acid colours provide tentative class-level annotations rather than definitive compound identities, and the manuscript has been revised to maintain that distinction. LC-MS, GC-MS or NMR would add compound-level identification, but the absence of these instruments does not invalidate the TLC, spectrophotometric or bioassay findings reported at their appropriate analytical level. Likewise, PCA or PLS would be unsuitable for four site-derived extracts and is not required for a descriptive two-provenance comparison.

The antioxidant results were not uniformly aligned by site. MP hexane performed best in the DPPH assay (IC50 = 0.19 mg/mL), while LP DCM performed best in hydrogen peroxide scavenging (IC50 = 0.48 mg/mL); reducing power estimates were similar (0.48–0.55 mg/mL). This inverse pattern is biologically informative because different assays probe different reaction mechanisms and different solvents enrich different constituent classes. It also cautions against attributing all activity to the higher flavonoid-equivalent value of one extract. With only four extracts, a statistical correlation between chemical measures and bioactivities would be unstable and potentially misleading.

The antibacterial findings require similar restraint. Qualitative dot plots and TLC bioautography showed putative active regions, but the broth microdilution endpoint detected activity only for DCM against *E. faecalis*, with the same MIC (0.50 mg/mL) for both sites. All other MICs exceeded 1.00 mg/mL. The quantitative antibacterial data therefore do not support site-specific superiority, and the active TLC regions require isolation and structural confirmation before being assigned to individual compounds [9,43]. Cytotoxicity and genotoxicity were not assessed and should be included in subsequent safety characterisation [45].

Rainfall, altitude, soil chemistry, temperature, light exposure and water stress are plausible contributors to variation in plant secondary metabolism [19,20,21,22,23,44,46]. Because these variables were not measured, they remain mechanistic hypotheses rather than demonstrated explanations for the Bushbuckridge-Mafarana differences. The unrecorded number of trees contributing to each pooled site sample also limits assessment of within-site variability. Broader follow-up should include multiple populations and seasons where feasible and should record relevant environmental and plant-level variables. Advanced compound characterisation would be valuable when access becomes available, but it is presented as a future extension rather than a condition for interpreting the current TLC and bioassay data.

## 5. Conclusions

Hexane and DCM extracts prepared from pooled *P. africanum* material collected at Bushbuckridge and Mafarana Village showed geographical provenance- and solvent-associated differences in phytochemical fingerprints and antioxidant responses. The MP hexane extract was the strongest candidate for DPPH-focused follow-up, whereas LP DCM was the strongest candidate in the hydrogen peroxide assay. In contrast, the MIC assay showed no between-site difference: only DCM inhibited *E. faecalis* within the tested range, at 0.50 mg/mL for both provenances.

The findings support the central claim that the two sampled provenances yielded distinguishable lipophilic extracts. They do not imply that one site represents all plant populations within its province, nor do they identify the environmental variable or individual compound responsible for each difference.

Collection locality should therefore be recorded during harvesting and considered during chemical quality control and bioactivity-guided selection. The present data justify assay-specific prioritisation of the tested extracts, while broader multi-site sampling and compound-level characterisation remain important extensions for province-wide standardisation.

## Figures and Tables

**Figure 1:**
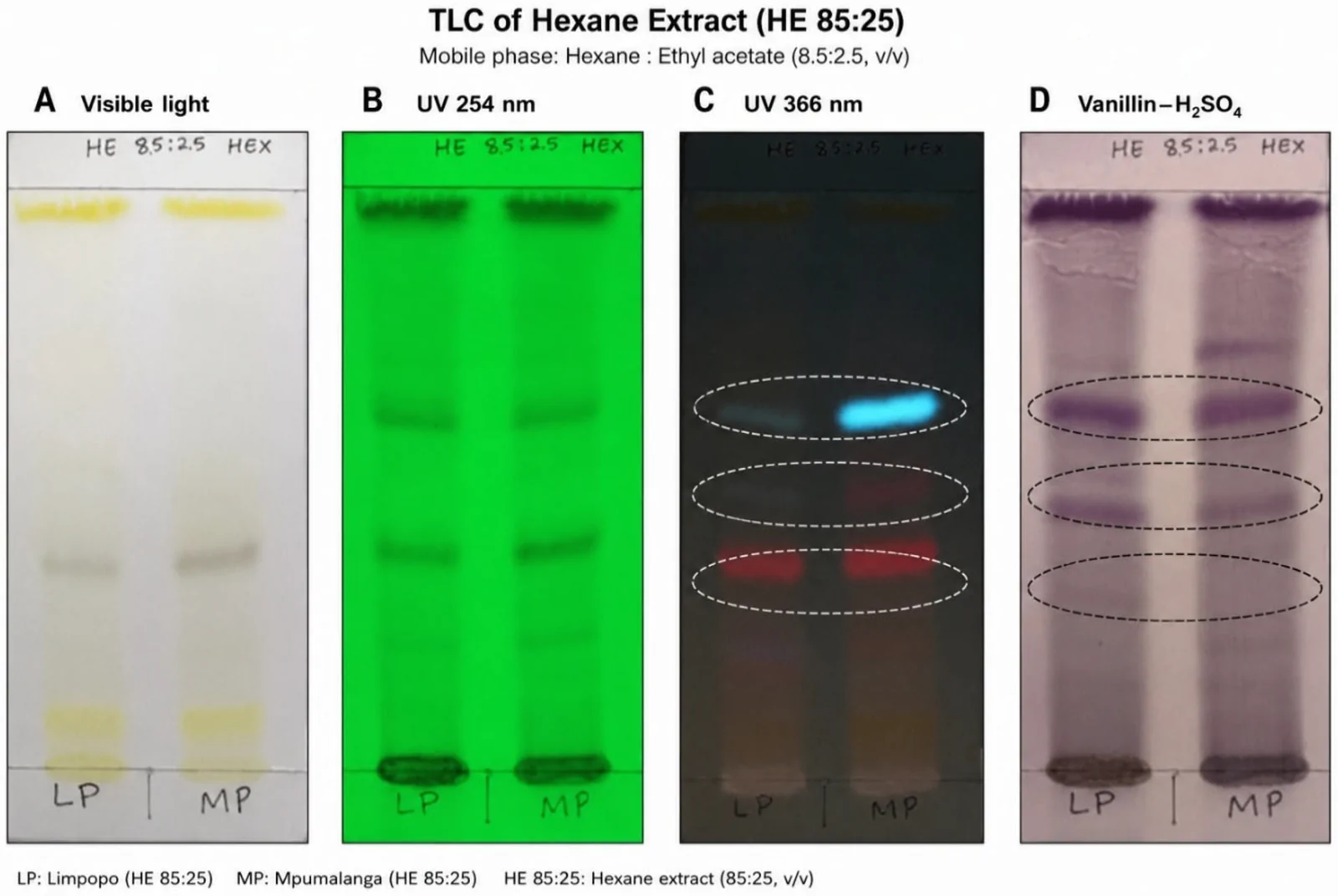
TLC fingerprint of hexane leaf extracts of *P. africanum* derived from material collected at Bushbuckridge, Mpumalanga (MP), and Mafarana Village, Limpopo (LP), developed in hexane:ethyl acetate (8.5:2.5, *v*/*v*). Chromatograms were visualised under (**A**) visible light, (**B**) UV 254 nm, (**C**) UV 366 nm and (**D**) after vanillin-sulphuric acid derivatisation. Circled regions highlight bands that differ in position or intensity between the two site-derived extracts.

**Figure 2:**
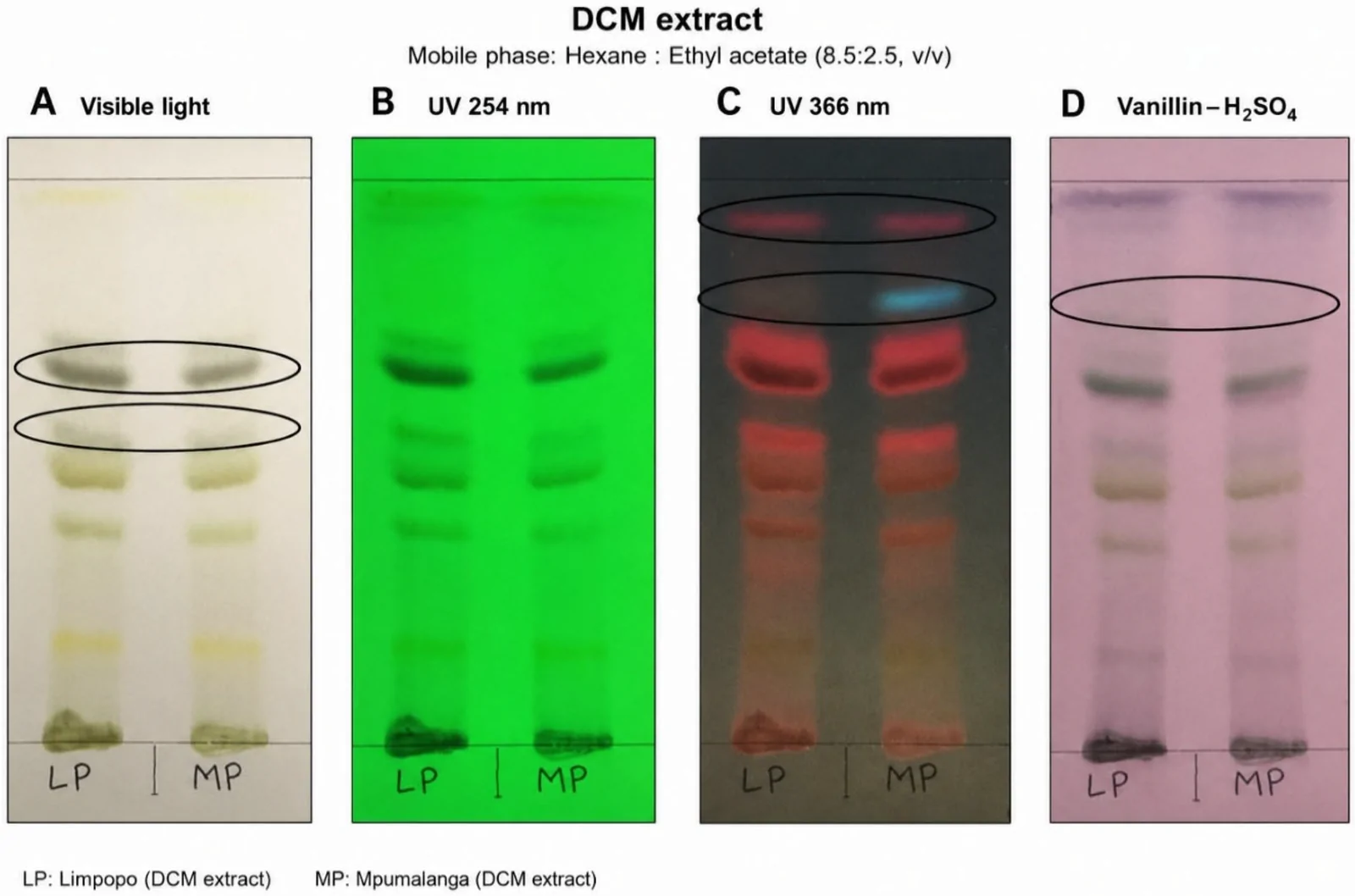
TLC fingerprint of dichloromethane (DCM) leaf extracts of *P. africanum* derived from material collected at Bushbuckridge, Mpumalanga (MP), and Mafarana Village, Limpopo (LP), developed in hexane:ethyl acetate (8.5:2.5, *v*/*v*). Chromatograms were visualised under (**A**) visible light, (**B**) UV 254 nm, (**C**) UV 366 nm and (**D**) after vanillin-sulphuric acid derivatisation. Circled regions highlight bands that differ in position or intensity between the two site-derived extracts.

**Figure 3:**
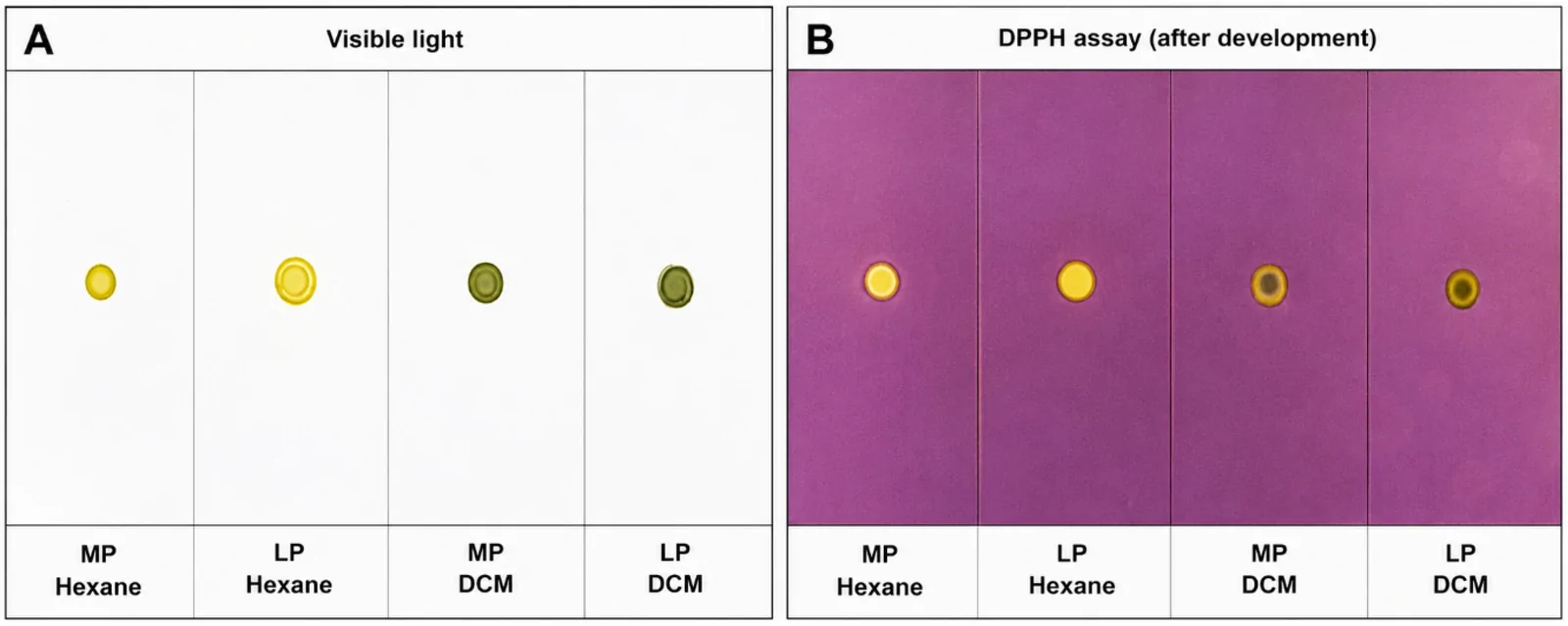
Qualitative DPPH dot-plot assay of hexane and dichloromethane leaf extracts of *P. africanum* derived from material collected at Bushbuckridge, Mpumalanga (MP), and Mafarana Village, Limpopo (LP). Yellow discolouration after DPPH application indicates radical-scavenging activity.

**Figure 4:**
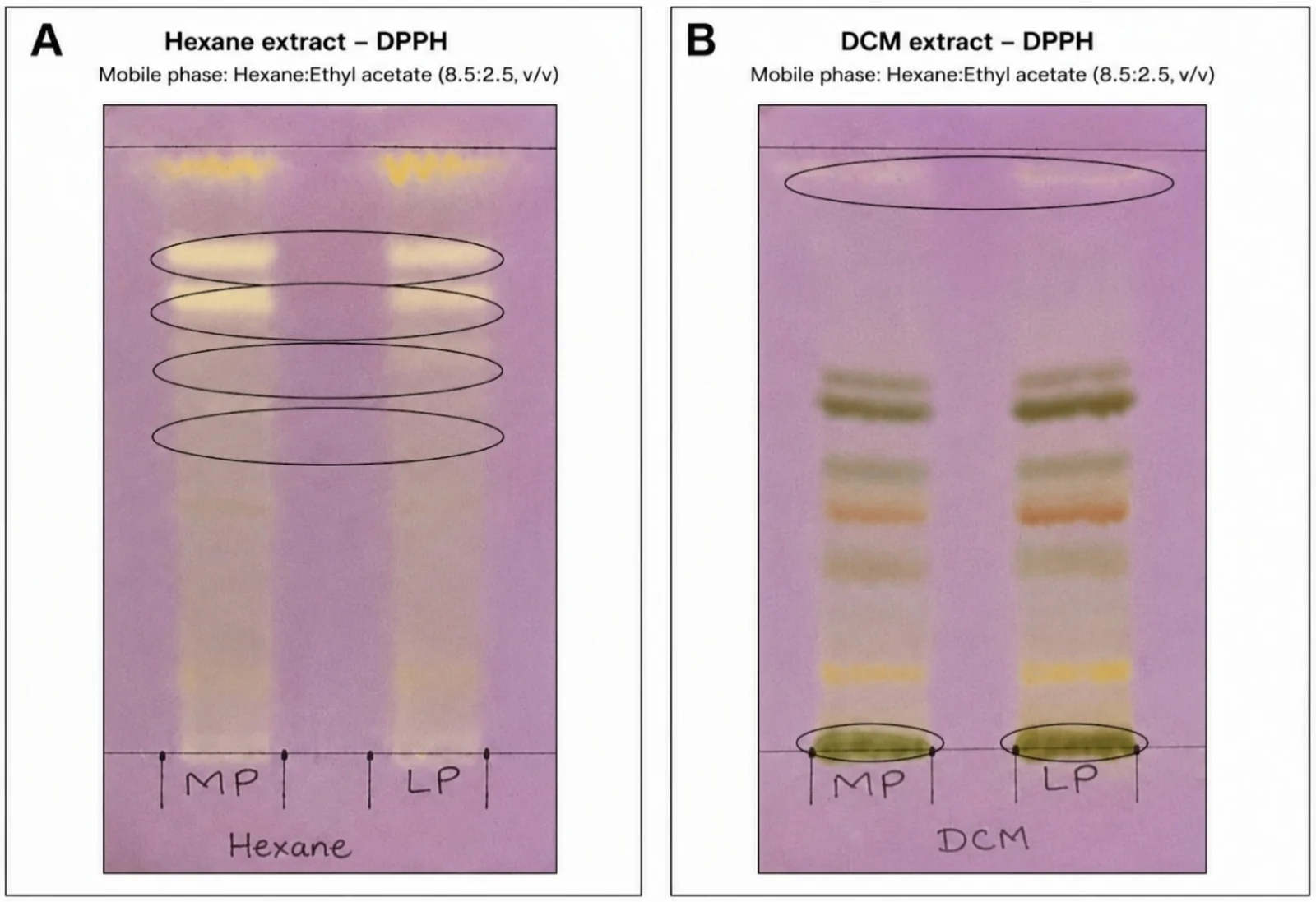
TLC-DPPH bioautography of (**A**) hexane and (**B**) dichloromethane leaf extracts of *P. africanum* derived from material collected at Bushbuckridge, Mpumalanga (MP), and Mafarana Village, Limpopo (LP), following development in hexane:ethyl acetate (8.5:2.5, *v*/*v*). Yellow bands against a purple background indicate radical-scavenging constituents. Circled regions highlight antioxidant-active bands.

**Figure 5:**
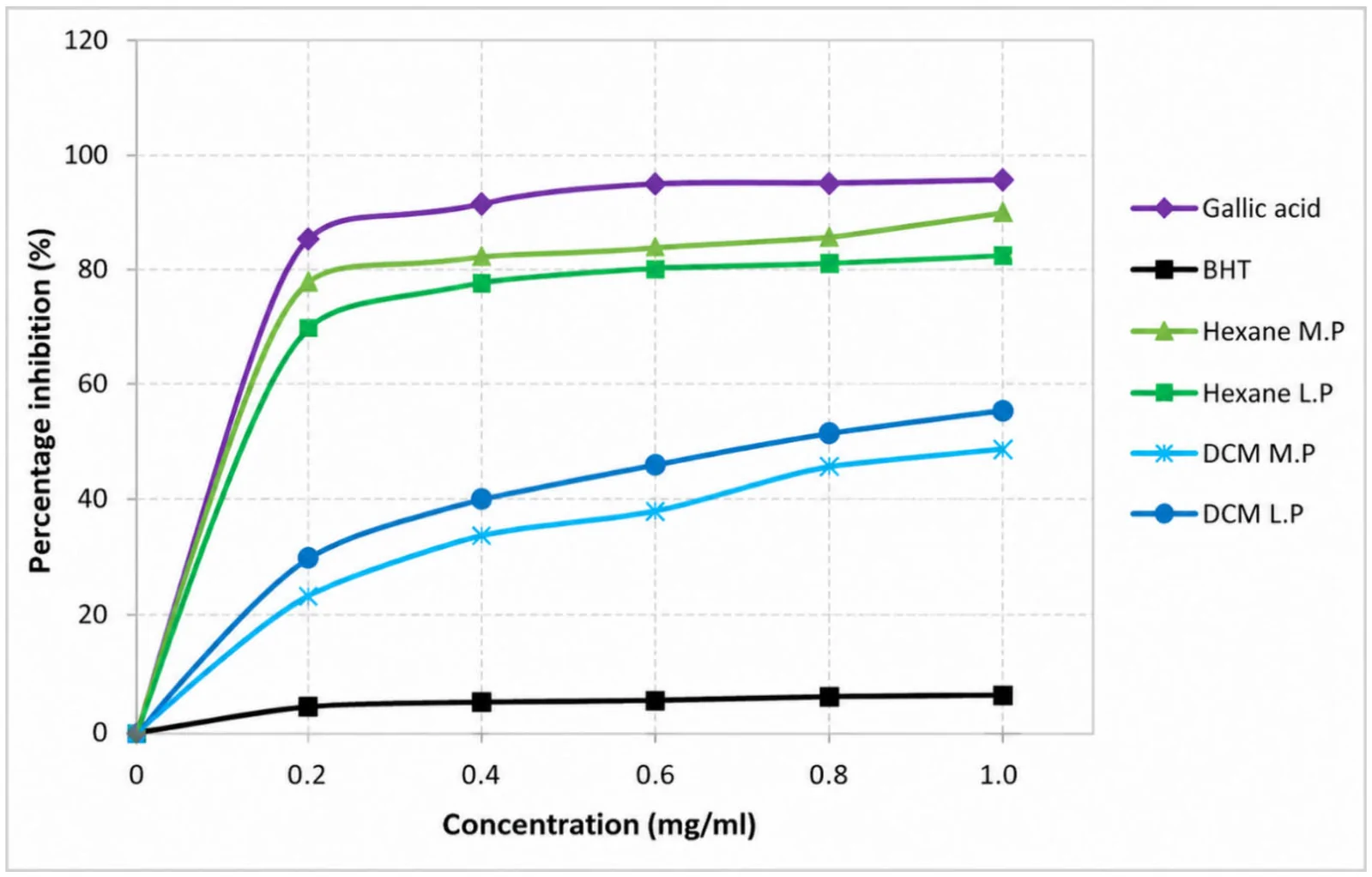
DPPH radical-scavenging activity of hexane and dichloromethane leaf extracts of *P. africanum* derived from material collected at Bushbuckridge, Mpumalanga (MP), and Mafarana Village, Limpopo (LP). Gallic acid and butylated hydroxytoluene (BHT) were reference antioxidants.

**Figure 6:**
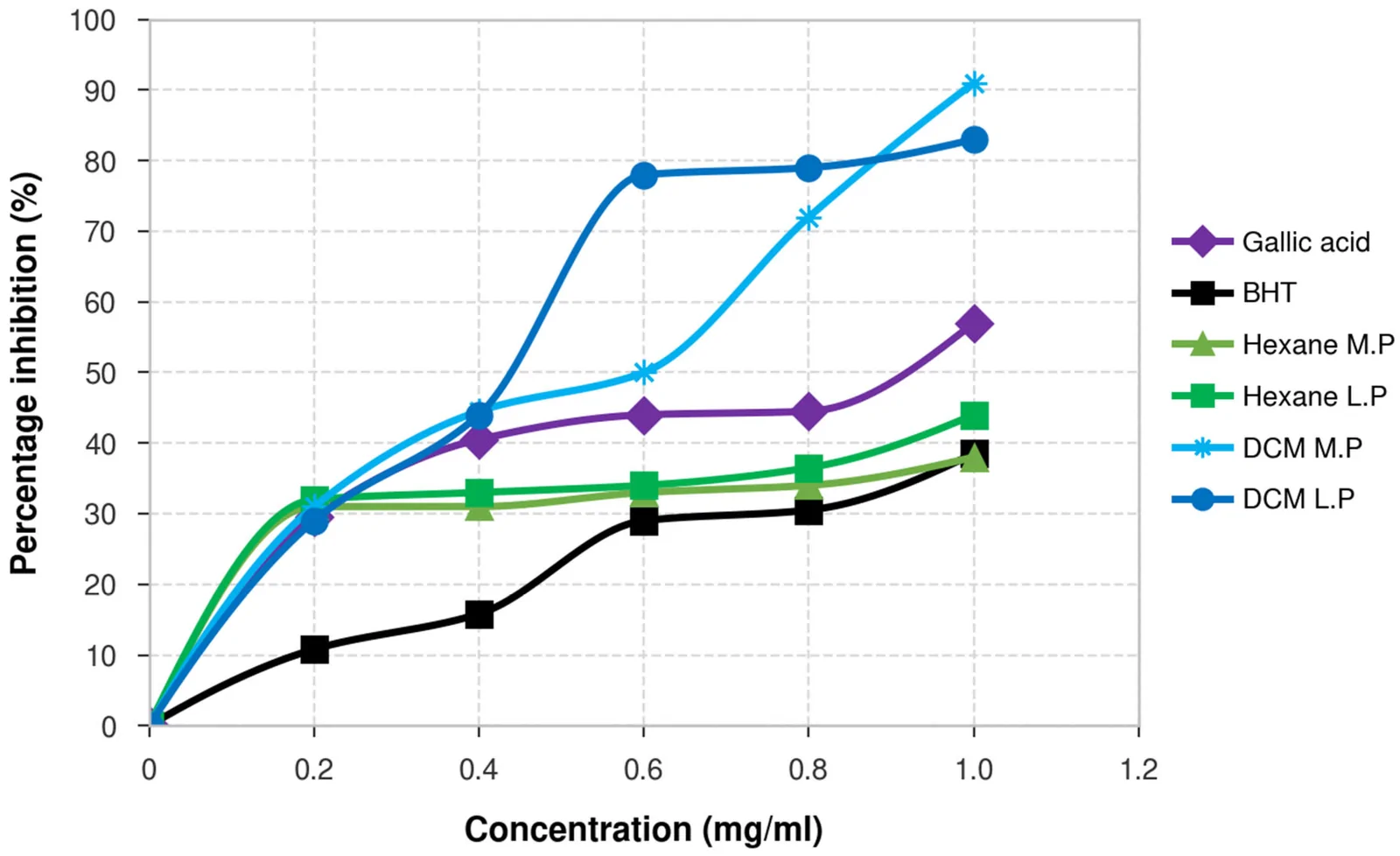
Hydrogen peroxide-scavenging activity of hexane and dichloromethane leaf extracts of *P. africanum* derived from material collected at Bushbuckridge, Mpumalanga (MP), and Mafarana Village, Limpopo (LP). Gallic acid and BHT were reference antioxidants.

**Figure 7:**
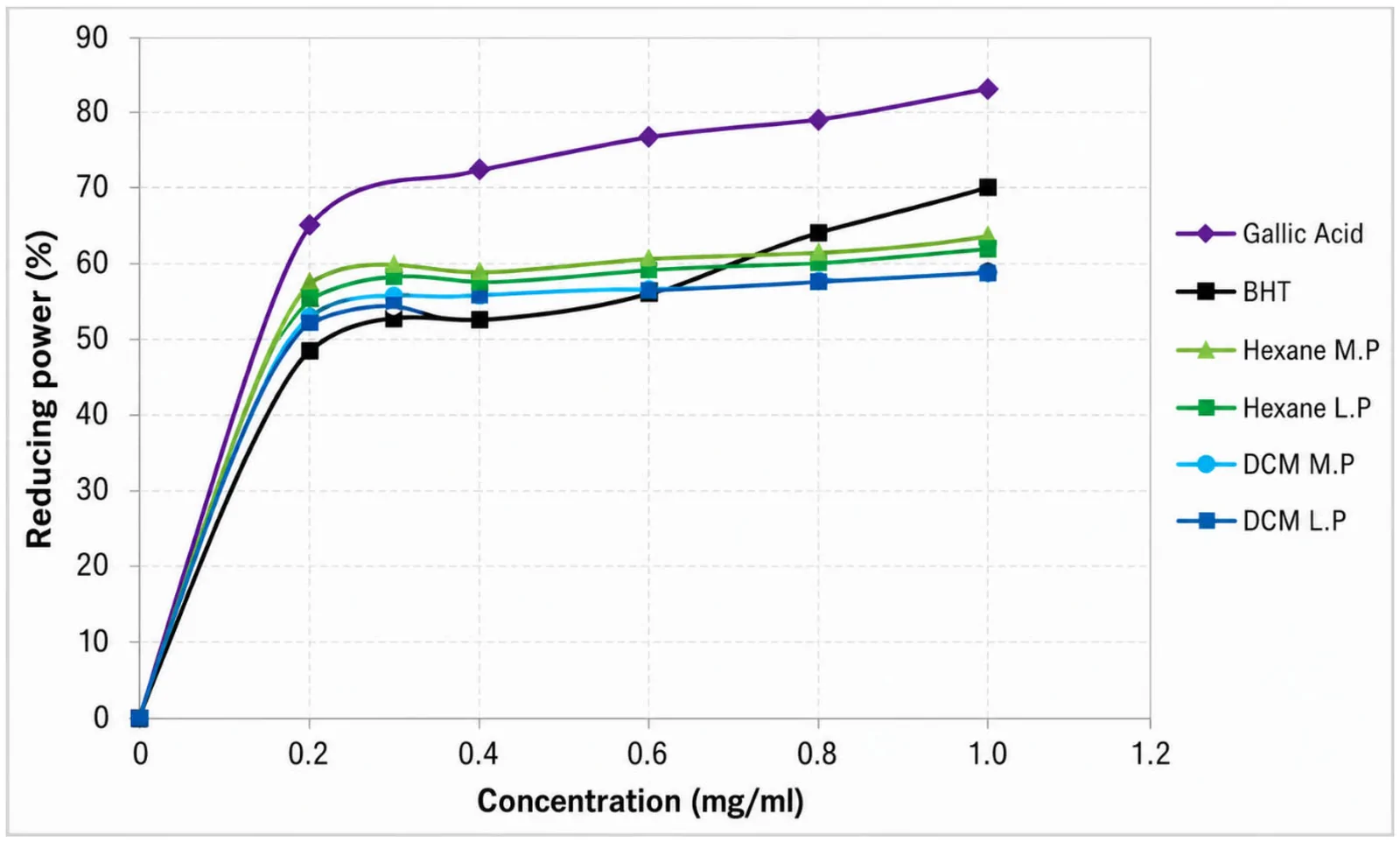
Ferric reducing power of hexane and dichloromethane leaf extracts of *P. africanum* derived from material collected at Bushbuckridge, Mpumalanga (MP), and Mafarana Village, Limpopo (LP). Gallic acid and BHT were reference antioxidants.

**Figure 8:**
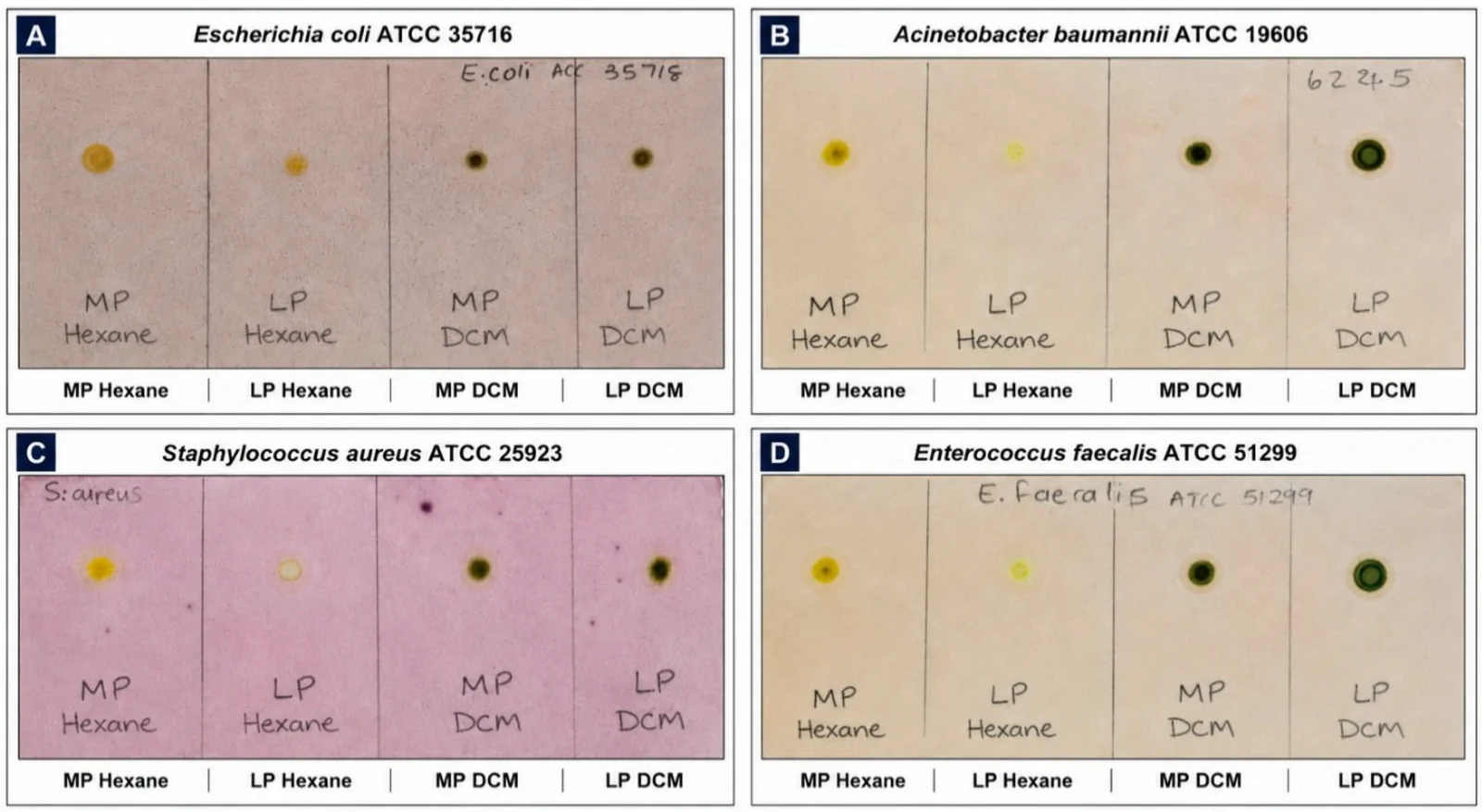
Qualitative antibacterial dot-plot screening of hexane and dichloromethane leaf extracts of *P. africanum* derived from material collected at Bushbuckridge, Mpumalanga (MP), and Mafarana Village, Limpopo (LP), against (**A**) *E. coli* ATCC 35716, (**B**) *A. baumannii* ATCC 19606, (**C**) *S. aureus* ATCC 25923 and (**D**) *E. faecalis* ATCC 51299.

**Figure 9:**
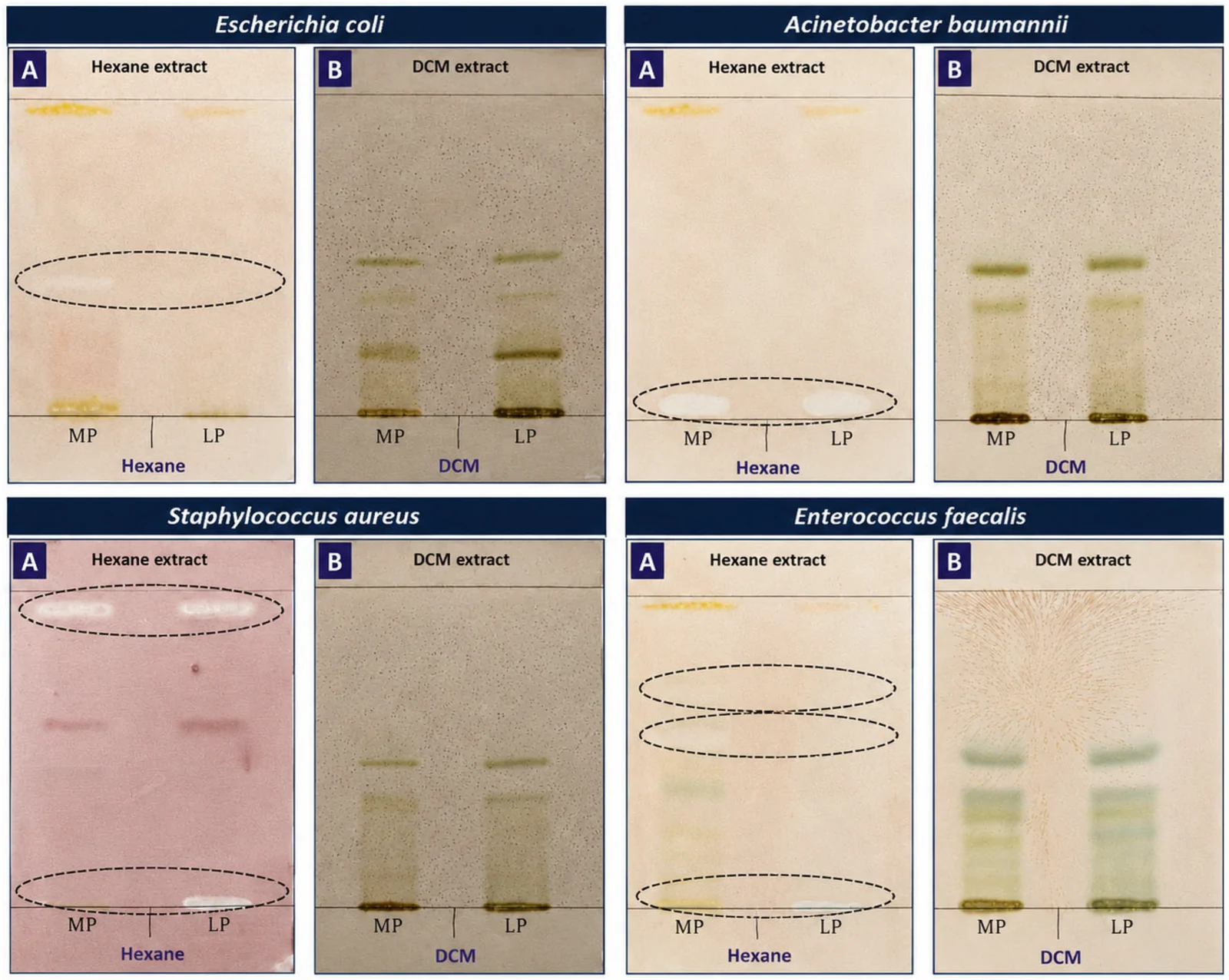
TLC antibacterial bioautography of hexane and dichloromethane leaf extracts of *P. africanum* derived from material collected at Bushbuckridge, Mpumalanga (MP), and Mafarana Village, Limpopo (LP), against *E. coli* ATCC 35716, *A. baumannii* ATCC 19606, *S. aureus* ATCC 25923 and *E. faecalis* ATCC 51299. Within each organism group, panel A shows the hexane extract and panel B the DCM extract. Clear zones after INT staining indicate chromatographic regions with growth-inhibitory activity. Circled regions highlight clear growth-inhibition zones.

**Table 1:** Qualitative distribution of phytochemical classes in hexane and dichloromethane leaf extracts of *P. africanum* derived from material collected at Bushbuckridge, Mpumalanga (MP), and Mafarana Village, Limpopo (LP).

Phytochemical	Hexane		Dichloromethane	
	MP	LP	MP	LP
Alkaloids	+	+	–	–
Anthraquinones	–	–	–	–
Cardiac glycosides	+	–	++	+
Flavonoids	±	+	±	–
Phenolic compounds	–	–	±	+
Tannins	–	–	±	±
Steroids	–	–	–	–

Legend: − = absent; ± = trace presence; + = moderate presence; ++ = abundant presence.

**Table 2:** Technical replicate summaries of selected phytochemical measurements in hexane and dichloromethane leaf extracts of *P. africanum* derived from material collected at Bushbuckridge, Mpumalanga (MP), and Mafarana Village, Limpopo (LP). Values are mean ± SD for technical measurements.

Phytochemical	Hexane		Dichloromethane	
	MP	LP	MP	LP
Saponins (mg DE/g extract)	297.38 ± 60.94	403.40 ± 22.94	1.30 ± 0.34	10.07 ± 10.59
Phenolic compounds (mg GAE/g extract)	ND	ND	ND	ND
Tannins (mg GAE/g extract)	3.74 ± 0.77	2.11 ± 0.10	2.28 ± 0.21	1.54 ± 0.33
Flavonoids (mg QE/g extract)	223.45 ± 16.74	136.00 ± 4.93	193.53 ± 88.72	155.12 ± 15.27

**Table 3:** Major TLC bands observed in the hexane and dichloromethane extracts of *P. africanum* developed in hexane:ethyl acetate (8.5:2.5, *v*/*v*) and visualised after vanillin-sulphuric acid derivatisation. Class assignments are tentative colour-based annotations and are not compound identifications (✓, present; −, absent).

Band	Extract	Rf	Colour (Vanillin)	MP	LP	Tentative Colour-Based Annotation
H1	Hexane	0.18	Purple	✓	✓	Terpenoid-like
H2	Hexane	0.42	Blue	✓	–	Flavonoid-like
H3	Hexane	0.63	Brown	–	✓	Phenolic-like
D1	DCM	0.29	Green	✓	✓	Unassigned
D2	DCM	0.81	Yellow	✓	–	Flavonoid-like

**Table 4:** Antioxidant IC50 point estimates (mg/mL) for hexane and dichloromethane leaf extracts of *P. africanum* derived from material collected at Bushbuckridge, Mpumalanga (MP), and Mafarana Village, Limpopo (LP).

Extract/Standard	DPPH		H_2_O_2_ Scavenging		Reducing Power	
	MP	LP	MP	LP	MP	LP
Hexane	0.19	0.24	>1.00 (1.26)	>1.00 (1.10)	0.50	0.48
DCM	0.81	0.74	0.52	0.48	0.54	0.55
Gallic acid	0.15	–	0.79	–	0.30	–
BHT	>1.00 (12.00)	–	>1.00 (1.27)	–	0.52	–

Values are IC50 point estimates derived from concentration-response data. Values in parentheses exceed the highest tested concentration (1.00 mg/mL) and should be interpreted as extrapolations. No site-level inferential comparison was performed.

**Table 5:** Minimum inhibitory concentrations (MIC, mg/mL) of hexane and dichloromethane leaf extracts of *P. africanum* derived from material collected at Bushbuckridge, Mpumalanga (MP), and Mafarana Village, Limpopo (LP), against selected bacteria.

Extract	*E. coli* MP	*E. coli* LP	*A. baumannii* MP	*A. baumannii* LP	*S. aureus* MP	*S. aureus* LP	*E. faecalis* MP	*E. faecalis* LP
Hexane	>1.00	>1.00	>1.00	>1.00	>1.00	>1.00	>1.00	>1.00
DCM	>1.00	>1.00	>1.00	>1.00	>1.00	>1.00	0.50	0.50

## Data Availability

The datasets generated and/or analysed during the current study are available from the corresponding author upon reasonable request. The data are not publicly available because they form part of an ongoing programme of research.

## Abbreviations

The following abbreviations are used in this manuscript:

ATCC	American Type Culture Collection
BHT	Butylated hydroxytoluene
DCM	Dichloromethane
DE	Diosgenin equivalents
DPPH	2,2-Diphenyl-1-picrylhydrazyl
GAE	Gallic acid equivalents
H_2_O_2_	Hydrogen peroxide
IC_50_	Concentration required to produce 50% inhibition
INT	*p*-Iodonitrotetrazolium violet
LP	Limpopo Province
MIC	Minimum inhibitory concentration
MP	Mpumalanga Province
QE	Quercetin equivalents
Rf	Retention factor
SANBI	South African National Biodiversity Institute
SD	Standard deviation
SMU	Sefako Makgatho Health Sciences University
SMUREC	Sefako Makgatho Health Sciences University Research Ethics Committee
TLC	Thin-layer chromatography
UV	Ultraviolet
